# Occupational exposure to vapor, gas, dust and fume using job-exposure matrix in a population-based, nationwide study in Kazakhstan

**DOI:** 10.1186/s12995-026-00501-z

**Published:** 2026-02-21

**Authors:** Denis Vinnikov, Irina Mukatova, Zhangir Dairuly, Aizhan Raushanova, Zhanna Romanova

**Affiliations:** 1 Al-Farabi Kazakh National University, al-Farabi avenue 71, Almaty, 050040 Kazakhstan; 2 Peoples’ Friendship University of Russia (RUDN University), Moscow, Russian Federation; 3 Astana Medical University, Astana, Kazakhstan

**Keywords:** occupational, airborne pollutant, JEM, population-based

## Abstract

**Background:**

We aimed to characterize the populational exposure to vapors, gases, dusts and fumes (VGDF) in a nationwide population-based study in Kazakhstan.

**Methods:**

Data on all positions held for more than a year in the lifetime were collected from 5058 (median age 47 (interquartile range (IQR) 31;61) years, 50% men) participants. Prevalence of each exposure was computed using ACE job-exposure matrix (JEM), and a multivariate logistic regression was applied to compute the odds ratios (OR) with 95% confidence intervals (CI) of such exposure.

**Results:**

Lifetime exposure to VGDF was found in 53% of general population, more in men (61% vs. 45%, *p* < 0.001), with dust (45%), mineral dust (32%) and gas (30%) being the top three exposures. Ever-smoking (OR 1.58; 95% CI 1.36;1.83), exposure to secondhand smoke (OR 1.49; 95% CI 1.29;1.71), education below university level (OR 2.12; 95% CI 1.86;2.41), being a male (OR 1.35; 95% CI 1.17;1.55), and living in the North and Center increased the odds of being exposed, independent of each other and work initiation since independence, body mass index and even the use of fossil fuel for heating.

**Conclusions:**

Exposure to VGDF in the general population of Kazakhstan is high, with some regional discrepancies. Predictors of such exposure can help identify most exposed population to tailor targeted preventive interventions.

**Supplementary Information:**

The online version contains supplementary material available at 10.1186/s12995-026-00501-z.

## Background

Kazakhstan is the 9th largest country in the world with very low population density and with a contrasting range of climatic, environmental and occupational conditions. The country exhibits high burden of chronic respiratory conditions, including chronic obstructive pulmonary disease (COPD) [1]. Studies explaining high burden of chronic respiratory conditions, including those quantifying smoking prevalence and determinants [2] are scarce; and high smoking prevalence [1, 2], high ambient and indoor air pollution [3] can explain most cases of high respiratory morbidity and mortality rates Occupational exposures, however, remain largely understudied. Given that the country remains in transition from the former highly industrialized economic model, many residents may report heavy exposure in the workplace to vapor, gas, dust and fume (VGDF) in the past, whereas young and middle-aged citizens may have low levels of exposure overall. However, there exist no nationwide studies depicting the level of population exposure in Kazakhstan, thus the contribution of occupational exposure in the chronic respiratory disease has yet to be elucidated.

In only one existing study which characterized the occupational profile of a sample of 1500 residents in Almaty, self-reported occupational history was used to ascertain exposures indirectly [1]. Although some options exist with regard to occupational questionnaires [4], these opportunities are usually missed in studies published from Central Asia, including Kazakhstan, and neither general, nor job-specific or even exposure-specific tools have been used to demonstrate exposure profile of a general population. Moreover, the agreement between assessment options may be poor [5]. Job-exposure matrices have gained popularity in the last decades [6] over self-reported exposure status because they can quite precisely identify exposed population, especially when the individuals may not fully recall their exposures in the workplace. Thus, these tools are now widely used on a populational level to classify exposure, including those to VGDF.

Given that percent of exposed population and regional differences in the exposure are essential for public health action with the purpose to allocate resources and estimate the occupational burden for most respiratory and cardiovascular diseases, our study was planned to fill the gap for Kazakhstan. We, therefore, aimed to quantify occupational exposure of the general population to VGDF in a nationwide, population-based study representing all major parts of the country.

## Methods

### Study design and sample

The study was approved by the Committee on Bioethics of al-Farabi Kazakh National University (approval IRB-A387, dated 17.03.2022), and all participants provided a written informed consent to participate. It was completed in compliance with the Declaration of Helsinki and local legislation on studies in humans. The study was designed as a population-based nationwide study, in which we included subjects from five major geographical parts of Kazakhstan [7]. We elected to divide the country into five locations (West, North, South, East and Center) and chose the largest city in each location as a study venue. Using such approach, we included Aktobe (West), Kostanay (North), Shymkent (South), Ust-Kamenogorsk (East) and Karagandy (Center).

The overall sample for five regions was set to 5000 participants aged 18 years and older, approximately 1000 from each region. Such sample allowed to achieve 97% power. Study participants were invited by nurses of the local outpatient medical facilities. The population covered by these medical facilities is a complete representation of the general population of those cities. Participants were invited to participate from the list of enlisted population using random numbers. They received a phone call from the medical center and then arrived there for an appointment and were asked to complete a questionnaire. Exclusion criteria were physical or mental limitations to fill the questionnaire in and age below 18.

### Questionnaire

The questionnaire was completed in an interviewer-assisted manner in Russian or Kazakh following the written informed consent. The first part included basic demographic information, such as full name, mobile phone, ID, date of birth, biological gender, residential address, ethnicity, highest attained education, and two questions reflecting the socioeconomic status. These included the estimated family income per person per month and a question on the highest attained education. A set of questions comprised occupational history, including all places of work in a lifetime and work duration in years. We only considered posts with the duration more than one year. Positions with a shorter duration were omitted.

A set of questions allowed to classify subjects into (a) never-smokers; (b) ex-smokers; (c) current occasional smokers, and (d) current daily cigarette smokers. Additionally, daily cigarette smokers provided information on the number of cigarettes smoked per day and smoking duration in years. Ex-smokers could provide data on the years of former smoking overall and the duration of abstinence in years. We also collected information on the electronic device use, vaping, and waterpipe smoking. For waterpipe smokers, we offered an additional question on smoking frequency per month. Finally, all subjects were asked to classify their exposure to environmental tobacco smoke (secondhand smoke) at home or in the workplace.

Because fossil fuel use for heating and even cooking is still prevalent in selected locations in Kazakhstan, we also considered exposure to woodsmoke or burning coal for heating at home and for cooking (any fuel other than natural gas or electricity). Regular physical activity was classified as positive if a person reported exercising at least three times a week for at least one hour.

### JEM-defined exposure to vapors, gases, dusts, fumes and mists

Because subjects may misclassify exposures in the workplace, especially those some time ago, we utilized airborne chemical job-exposure matrix (ACE JEM) to convert all positions held in the lifetime for more than a year to VGDF [8]. In the population-based studies, the use of JEMs may be beneficial over self-reported exposures, although concordance between these two approaches may be limited [6, 9, 10]. ACE JEM has been reported as a reliable instrument to develop information on the occupational exposure via inhalational route from the UK Biobank [8]. We identified each position held for a year or more, linked it to the Standard Occupational Classification (SOC) 2000 codes and then converted them to occupational exposure to either vapor, gas, dust, biological dust, mineral dust, fume, diesel exhaust, fiber, mist, asbestos, metal and a composite exposure to VGDF. The tool allows to classify exposure to VGDF as a binary variable, then define percent exposed population for each SOC code and additionally the average level of exposure (Additional File 1). We only classified jobs into with exposure or without exposure to VGDF and then computed the overall years in service for all positions defined as VGDF-associated. Coding occupations from the questionnaires was performed by an occupational doctor to minimize misclassification.

### Statistical analysis

The primary outcome of interest in this study was the overall exposure rate in the studied population aged 18 years and older, as a percent of exposed overall and in the selected groups. Testing data for normality using Shapiro-Wilk test showed non-normal distribution of most data in the dataset; therefore, we applied non-parametric tests in all comparisons. We characterized continuous variables with medians and their corresponding interquartile ranges (IQR), and binary variables were shown as percent to the whole group. Non-parametric tests to compare two groups were Mann-Whitney U-test or χ2 tests for binary data. We described percent exposed first for the whole group, then in men vs. women; residents of five regions with each other and a few other univariate comparisons, including with a university degree vs. those without, ever-smoking vs. never-smoking; exposed to secondhand smoke vs. none-exposed; and fossil fuel users for heating with those who use gas or central heating. Continuous variables in such univariate comparisons included age and body mass index (BMI).

Variables significantly associated with exposure to VGDF (*p* < 0.05) using χ^2^ tests, were then included in the adjusted for each other multivariate model of logistic regression, in which we reported adjusted odds ratios (OR) with the corresponding 95% confidence intervals (CI). -Since Soviet Union disruption was one of the main drivers of the economic change in Kazakhstan, we have elected to apply a binary variable “started working after 1992” instead of a continuous age variable in a multivariate logistic regression. Adjusted model effects of each included predictor reflected their independent of other predictors contribution to the overall effect. In all tests, p-values below 0.05 were considered significant, and all tests were accomplished in NCSS 2025 (Utah, USA).

## Results

Our sample included 5058 subjects, aged 47 (IQR 31;61) years living in five large cities of the country representing all its major five parts. High school and higher education levels were reported by 99% of the population (Table 1). Almost 40% of the sample were ever-cigarette smokers, and 23% were smoking cigarettes daily. About one-third of the population were exposed to secondhand smoke either at home or at work. We found contrasting picture of exposure to secondhand smoke, similar to smoking status in men compared to women, and the former were more exposed. In addition, men in this study were younger, had lower BMI and showed lower fraction of subjects with the university degree or higher compared to women.

**Table 1:** Demographic, lifestyle and exposure data of study participants

Variable	Overall, *N* (%)	Exposed, *N* (%)	Non-exposed, *N* (%)
N (%)	5058 (100)	2677 (53)	2381 (47)
Male sex*	2548 (50)	1554 (58)	994 (42)
Age, years*	47 (31;61)	49 (35;60)	45 (25;61)
BMI, kg/m^2^*	26 (22.7;29.8)	26.2 (23.2;29.8)	25.8 (22.3;29.7)
Highest attained education*			
Secondary school	64 (1)	43 (2)	21 (0)
High school	1541 (30)	750 (28)	791 (33)
College	1797 (36)	1212 (45)	585 (25)
University degree	1615 (32)	667 (25)	948 (40)
Academic degree	41 (1)	4 (0)	37 (2)
Cigarette smoking status, N (%)*			
Never-smoker	3145 (62)	1433 (53)	1712 (72)
Former smoker	722 (14)	441 (17)	281 (12)
Daily smoker	1160 (23)	781 (29)	379 (16)
Occasional smoker	31 (1)	22 (1)	9 (0)
Waterpipe smoking, N (%)	193 (4)	107 (4)	86 (4)
Exposure to secondhand smoke, N (%)*	1504 (30)	966 (36)	538 (23)

Note: * - *p* < 0.05 in either Mann-Whitney U-test for continuous variables or χ2-test for categorical variables; VGDF – vapors, gases, dusts and fumes

Overall, more than half of the sample were exposed to VGDF for more than a year in their lifetime, and significantly more men exposed in the workplace compared to women. Such exposure lasted on average for 13 years, also more in women. Spearman’ s correlation between age and years of exposure to VGDF in those exposed was 0.62 (95% CI 0.60;0.65). Most exposed population resided in the North, whereas the least exposed was South (Table 2). Three most prevalent exposures of VGDFs were dust (45%), mineral dust (32%) and gas (30%), followed by asbestos (29%), vapor (28%), fume (26%), biological dust (24%), diesel and mist (19% each) and fiber (27%). The least prevalent exposure was to metal (8%). Kazakhstan regions differ from each other significantly in the precent exposed population with regard to specific exposure (Table 2). Asbestos was the only evenly distributed airborne exposure, reported in about 30% of population.

**Table 2:** VGDF estimates for specific locations in the study

Exposure	West (Aktobe), *N* (%)	Center (Karaganda), *N* (%)	North (Kostanay), *N* (%)	East (Ust-Kamenogorsk), *N* (%)	South (Shymkent), *N* (%)
All VGDF*	473 (48)	541 (60)	783 (63)	492 (53)	388 (39)
Vapor*	351 (35)	196 (22)	343 (28)	220 (24)	288 (29)
Gas*	327 (33)	284 (31)	476 (38)	258 (28)	196 (20)
Dust*	449 (45)	452 (50)	609 (49)	408 (44)	343 (34)
Biological dust*	270 (27)	238 (26)	295 (24)	183 (20)	207 (21)
Mineral dust*	265 (27)	363 (40)	469 (38)	293 (32)	212 (21)
Fume*	220 (22)	270 (30)	488 (39)	256 (28)	102 (10)
Diesel*	185 (19)	182 (20)	359 (29)	154 (17)	84 (8)
Fiber*	190 (19)	164 (18)	311 (25)	142 (15)	62 (6)
Mist*	182 (18)	129 (14)	236 (19)	196 (21)	204 (20)
Asbestos	274 (28)	250 (28)	375 (30)	292 (32)	277 (28)
Metal*	36 (4)	81 (9)	178 (14)	108 (12)	14 (1)

Note: data are presented as the number of exposed population and percent exposed in brackets; * - p-value below 0.001 from χ^2^ test for a 2*5 table

Lifetime exposure to VGDF in the Kazakhstan population was not only associated with sex (Table 1) and region (Table 2), but also with ever-smoking (65% exposure in ever-smokers vs. 46% exposed in never-smokers, *p* < 0.001), age (median age in the exposed 49 years vs. 45 years in non-exposed, *p* < 0.001), education (41% exposed in those have a university degree or higher vs. 59% in those with no university degree, *p* < 0.001), exposure to secondhand smoke (64% of exposed to secondhand smoke vs. 48% in non-exposed to secondhand smoke, *p* < 0.001), and even the use of fossil fuel for heating (65% were exposed to VGDF in those using such fuel vs. 52% in non-users, *p* < 0.001) and BMI (median 26.2 in the exposed vs. 25.8 in non-exposed, *p* < 0.01). When all these variables were tested in the multivariate model, ever-smoking, exposure to secondhand smoke, poorer education, being a male, BMI and living in the North and Center increased the odds of being exposed to VGDF, while living in the East and South reduced the odds, all independent of each other (Table 3). The use of fossil fuel for heating and start working in 1992 were not associated with the outcome.

**Table 3:** Logistic regression, reporting ORs for being exposed to VGDF

Predictor	Crude OR (95% CI)	Adjusted OR (95% CI)
Start working in 1992	0.80 (0.72;0.90)	0.88 (0.77;1.00)
Sex, male	1.93 (1.73;2.16)	1.35 (1.17;1.55)
Ever-smoking	2.22 (1.98;2.50)	1.58 (1.36;1.83)
Education below university level	2.11 (1.87;2.38)	2.12 (1.86;2.41)
Exposure to secondhand smoke	1.93 (1.71;2.19)	1.49 (1.29;1.71)
Use of fossil fuel for heating	1.72 (1.28;2.30)	1.21 (0.89;1.66)
BMI	1.01 (1.00;1.02)	1.02 (1.01;1.03)
North, West, East, Center vs. South (ref)	2.06 (1.79;2.38)	1.80 (1.55;2.10)

Note: in the adjusted model, effects are adjusted for all shown variables; OR – odds ratio; CI – confidence interval

## Discussion

This population-based study is the first report on the prevalence and the structure of exposure to VGDF across the cities in Kazakhstan. It clearly confirms high overall proportion of exposed population in the country based on indirect measurements with a JEM. Depending on the region, from 39 to 63% of general population have ever been exposed to VGDF for more than a year in their lifetime. Such exposure is strongly and independently associated with cigarette smoking, educational level below university, exposure to secondhand smoke and being a male. Despite some limitations for JEM use, these data are a valuable pool of information to help plan public health interventions and allocate funds to mitigate the associated health effects of occupational exposures.

The proportion of exposed population is an important public health measure of the contribution of occupational exposures to a wide range of health effects, including, but not limited to, respiratory (COPD, asthma), cardiovascular (acute coronary syndrome, arrythmias) effects. Prior to this study, these data were unavailable for Kazakhstan, and for the entire region of Central Asia, and limited studies existed elsewhere. Those few studies reported the prevalence from 25% [11] − 33% [12] to 57% [13], but often reported prevalence fell within the range of 40–50% as in the Spanish [14], Norwegian [15], Danish [16, 17] and New Zealand [18] studies. Most of these studies have been designed to ascertain the risk of various outcomes, most often respiratory ones, with exposure to VGDF, and may be heterogenous in terms of the tools used for exposure classification. Depending on the methods to quantify exposure, the estimated proportion of exposed population may differ to a large extent. This creates some uncertainty in the estimates of the risks of the outcomes of interest. Nevertheless, the estimated proportion of exposed population in Kazakhstan is somewhat comparable to other countries and reflects the current economic model. Our current data now assume that half of the population are at risk of COPD, asthma and cardiovascular effects usually associated with VGDF.

One of the findings in this study was the regional disparity in the exposure levels. Kazakhstan is a country which inherited a huge economic infrastructure from the Soviet Union, but most industries of those times terminated, leaving out millions of people exposed in the past but no exposure at present. Significantly lower proportion of exposed people in the South now reflects the new economic reality in the country, since Shymkent in Soviet times was the center of chemical industry in the Republic, concentrating more than 100 large producers in one city, including large-scale phosphorus, polymetallic, cement, tyre, petrochemical, pharmaceutical and other industries. Discontinuation of large industrial production in the South since independence has now resulted in a significant drop in the percent of exposed population, elucidated in our analysis. Unlike other regions, population in the south is mostly employed by the government sector and private agriculture, whereas still operational large-scale industry in the northern, eastern and central regions sustains high fraction of exposed population.

The approach to estimate occupational exposures still remains the matter of scientific debate, as there exists no consensus on whether JEMs should be explicitly or exceptionally used in the population-based studies. Although JEMs remain a time- and cost-efficient approach to obtain estimates of occupational exposure [6], the source information for them are not always measurements, but often surveys, industry databases, and even unpublished reports [6], which are then synthesized in an expert report [10]. Therefore, the agreement between self-reported and JEM-defined exposure may be modest [9]. JEMs are more objective measures of exposure and tend to reduce the risk of recall bias. Particularly patients experiencing symptoms at work are prone to overestimate their exposure. Nevertheless, the JEM cannot take into consideration the differences among workers within the same occupation, which can lead to non-differential misclassification. Even for each specific exposure, an array of JEMs now exist [10], but they are not always coherent in the estimates [10]. Altogether, this creates some uncertainty in interpreting the rates of exposure obtained via JEMs. We believe that the high estimates we obtained in our study can thus not always be accurate, but comparison between geographical locations may be quite useful for public health policy.

Percent of exposed population is an important public health measure because it allows to calculate the population attributable fraction for VGDFs for a given outcome. VGDF are most studied for chronic respiratory conditions, predominantly COPD, in which such fraction can reach 14%, but may be lower in selected countries, like Kazakhstan [19]. With high smoking prevalence and such high estimated levels of occupational exposures, Kazakhstan is in the growing phase of societal burden of COPD [20], and huge costs to manage these patients should be considered for the future. Furthermore, health-related quality of life exhibits a clear association with occupational exposures [21, 22]; therefore, public health action in addressing health-related quality of life should also rely on the occupational exposure profile of the general population. These data can help set up a national plan on the mitigation of adverse negative effects of airborne exposures when the data on the prevalence and structure are now available.

Despite being based on a large sample, representing all five major locations, this study has distinct limitations. Firstly, we could not collect data from the rural regions due to organizational perils. Secondly, we limited this analysis to JEM-defined exposures only and could not consider self-reported exposure to VGDF in the workplace and thus could not compare their coherence. Thirdly, JEM used in this study was developed to investigate causes of COPD and to be applied to the UK Biobank data, for the period following the introduction of SOC 2000. Considering differences in exposures and jobs between United Kingdom and Kazakhstan, some exposure misclassification is likely. Because there have been no JEMs developed for Kazakhstan jobs and exposures, we expect some inaccuracy in the reported effects. All that necessitates future studies in Kazakhstan for the local context and population occupational profile. Moreover, other JEMs could be more helpful for estimating risk of asthma (asthma-specific JEM or updated OAsJEM) and cardiovascular diseases [23]. Fourthly, although response rate during population selection recruitment was reported very high by the polyclinic nurses, selection bias cannot be completely ruled out. Finally, we could not consider the use of personal protective equipment by the participants as this may have served a significant confounder.

Our results should be interpreted in the current context of occupational medicine both in Kazakhstan and the remaining four countries of the former Soviet Union in Central Asia, given that they all inherited a similar economic infrastructure. Difficulties, however, may arise in direct comparisons, because, on the one hand, we could not identify similar estimates in those countries in the biomedical literature. In the other hand, economic models of these countries have evolved into their specific structures, in which heavy industry remained in only a few, whereas other elected to transfer to a greater fraction of agriculture or sales. Therefore, such comparisons between countries are now challenging and may be misleading. Future studies from the neighboring countries should consider the changes in economic models.

## Conclusions

To conclude, this population-based study sheds light to the current structure and proportion of occupational exposure to VGDF in five major parts of Kazakhstan. About half of the population in the country may have been exposed to VGDF in their lifetime, but there is a notable geographical disparity in such exposure. Cigarette smoking, poorer education, exposure to secondhand smoke and being a male significantly increase the odds of being exposed. These data will help shape the plan of public health policy in the country with regard to modifiable risk factor of some respiratory and cardiovascular diseases, associated with work.

## Supplementary Information

Below is the link to the electronic supplementary material.

Supplementary Material 1

## Data Availability

The datasets used and/or analysed during the current study are available from the corresponding author on reasonable request.

## Abbreviations

ACE JEM: Airborne chemical job-exposure matrix
BMI: Body mass index
CI: Confidence interval
COPD: Chronic obstructive pulmonary disease
IQR: Interquartile range
JEM: Job-exposure matrix
OR: Odds ratio
SOC: Standard Occupational Classification
VGDF: Vapor, gas, dust and fume
