# Supplementary material for: Occupational exposure to vapor, gas, dust and fume using job-exposure matrix in a population-based, nationwide study in Kazakhstan
Source: J Occup Med Toxicol. 2026 Feb 21;21:13. doi: 10.1186/s12995-026-00501-z (PMC13032510; doi:10.1186/s12995-026-00501-z)
Supplement: Supplementary file 1 — Supplementary Material 1 [file 12995_2026_501_MOESM1_ESM.docx]

Supplementary Table 1. Examples of job classification using ACE JEM

| Job | Vapor | Gas | Dust | Bio-dust | Mineral dust | Fume | Diesel | Fiber | Mist | Asbestos | Metal | VGDF |
| --- | --- | --- | --- | --- | --- | --- | --- | --- | --- | --- | --- | --- |
| Chemical engineer | 1 | 1 | 1 | 0 | 1 | 0 | 0 | 0 | 1 | 1 | 0 | 1 |
| Medical practitioner | 1 | 1 | 1 | 1 | 0 | 0 | 0 | 0 | 0 | 1 | 0 | 1 |
| Architect | 0 | 0 | 0 | 0 | 0 | 0 | 0 | 0 | 0 | 0 | 0 | 0 |
| Graphic designer | 1 | 0 | 0 | 0 | 0 | 0 | 0 | 0 | 1 | 1 | 0 | 1 |
| Farmer | 0 | 1 | 1 | 1 | 0 | 1 | 1 | 1 | 1 | 1 | 0 | 1 |

Note: VGDF – vapor, gas, dust, fume
